# Exclusive breastfeeding practice during first six months of an infant’s life in Bangladesh: a country based cross-sectional study

**DOI:** 10.1186/s12887-018-1076-0

**Published:** 2018-03-02

**Authors:** Murad Hossain, Ashraful Islam, Tunku Kamarul, Golam Hossain

**Affiliations:** 1 Department of Orthopaedic Surgery, Tissue Engineering Group (TEG), National Orthopaedic Centre of Excellence in Research and Learning (NOCERAL), Faculty of Medicine, University of Malaya, 50603 Kuala Lumpur, Malaysia; 2 Department of Biotechnology and Genetic Engineering, Faculty of Science, Noakhali Science and Technology University, Noakhali-3814, Bangladesh; 3 Research Management Centre, Faculty of Medicine, University of Malaya, 50603 Kuala Lumpur, Malaysia; 4 University Malay Medical Center, 50603 Kuala Lumpur, Malaysia; 5 Department of Statistics, Faculty of Science, Rajshahi University, Rajshahi, 6205 Bangladesh

**Keywords:** Exclusive breastfeeding, Prevalence, Factors, Logistic regression, Bangladesh

## Abstract

**Background:**

Breastfeeding offers incredible health benefits to both child and mother. It is suggested by World Health Organization that an able mother should practice and maintain exclusive breastfeeding for first six months of her infant’s life. The objective of this study was to determine the prevalence and factors associated with exclusive breastfeeding for first six months of an infant’s life in Bangladesh.

**Methods:**

Data was extracted from Bangladesh Demographic and Health Survey (BDHS-2014). BDHS-2014 collected data from 17,863 Bangladeshi married women in reproductive age from the entire country using two stages stratified cluster sampling. We included only mothers having at least one child currently aged not less than 6 months. Mothers who did not have child to breastfeed, some incomplete information and missing samples were excluded from the data set and consequently 3541 mothers were considered in the present study. Chi-square test, binary logistic regression models were used in this study.

**Results:**

The prevalence of exclusive breastfeeding (EBF) for first six months of an infant’s life in Bangladesh was 35.90%. Binary multivariable logistic regression model demonstrated that relatively less educated mothers were more likely to exclusively breastfeed their children than higher educated mothers.

(AOR = 2.28, 95% CI: 1.05–4.93; *p* < 0.05). Housewife mothers were more likely to be EBF than their counterparts (AOR = 1.20, 95% CI: 1.02–1.42; *p* < 0.05). Higher rate of EBF was especially found among mothers who were living in Sylhet division, within 35–49 years old, and had access to mass media, had more than 4 children, had delivered at home and non-caesarean delivery, took breastfeeding counseling, antenatal and postnatal cares.

**Conclusions:**

Stepwise regression model exhibited that most of the important predictors were modifiable factors for exclusive breastfeeding. Authorities should provide basic education on EBF to educated mothers, and organize more general campaign on EBF.

## Background

Breast milk is the best source of nutrition to offer to the newborn babies which is uniquely tailored to meet all the nutritional needs of human babies for the first six months of life [1]. The nutrients of the breast milk are present in proper balance and are provided in bio-available and easily digestible forms [2]. It also possesses remarkable immunological and anti-inflammatory properties that protect both mothers and children against various infections and diseases [3]. Hence, breastfeeding is considered as one of the most important factors for growth and development of infants. Breastfeeding offers tremendous health benefits to both child and mother. Breastfeeding protects the infants against allergies, sickness and obesity [4]; at the same time it reduces the risk of having childhood infections e.g. ear infections and diseases e.g. diabetes and cancer [4, 5]. Breastfeeding also causes no constipation, diarrhea or stomach upset in infants [4]; it decreases postnatal mortality rates [6]. It can help to improve cognitive and motor development [7] and decreases the rates of sudden infant death syndrome [8]. Maternal benefits include: reduced risk of developing type 2 diabetes, ovarian and breast cancers [1, 4], lactational amenorrhea which could be a natural birth control [1, 9] and adequate weight recovery [1, 4, 9]. Besides health benefits, breastfeeding also ensures many other benefits that include economical, environmental and psychosocial benefits [10]. Moreover, breastfed children have been shown to possess higher intelligence quotient (IQ) [11]. For receiving optimum benefits, breastfeeding should be initiated within one hour after the birth of the infant and should be maintained exclusively for the first six months of the infant’s life. Exclusive breastfeeding (EBF) means that the newborn infant is fed only breast milk and no other liquids (not even water) or solids are given, with the exception of oral rehydration salt solution, vitamins, mineral supplements or medicines [11, 12]. The World Health Organization (WHO) recommended that an able mother should practice and maintain exclusive breastfeeding for first six months of her infant’s life [11, 12]. An effective EBF coverage has been estimated to avert 13%–15% of deaths among children under five years of age especially in middle and low earning settings [13]. Some researchers reported that children who received EBF were in lower risk of having acute respiratory and gastrointestinal infections compared to children who did not receive EBF [14, 15]. EBF is anticipated to prevent 13% of child deaths in high HIV prevalence settings [6]. It has also been shown that the rate of HIV transmission from mother to child is lower in exclusively breastfed children compared to non-EBF children [16].

Although EBF is vital to promote infants’ growth, development and health, however, globally only 50% of infants under 1 month of age and 30% of infants aged between 1 to 5 months are exclusively breastfed [17]. According to WHO’s report on early initiation and exclusive breastfeeding (2011), an overall prevalence of EBF was 36% globally, whereas the lowest rates of EBF were reported in West/Central Africa (20%) and the highest rates of EBF were found in East Asia/Pacific (43%) [18]. The EBF determining factors have been shown to vary between countries and within the same country as well. Previous studies have indicated several factors that are involved in determining EBF: educational level, occupation, knowledge on breastfeeding, breastfeeding counseling during antenatal care (ANC), infant feeding counseling during postnatal care (PNC), intent to exclusively breastfeed before delivery, attitudes towards EBF, timely initiation of breastfeeding, mothers’ smoking status, monthly household income, type of delivery, place of delivery, infant’s age and weight, residence, socio-economic position, parity, prelacteal feeding, discarding colostrums, community beliefs, health system practices and mothers’ HIV status [19–23].

In Bangladesh, the trend of practicing EBF among the lactating mothers remained mostly unchanged for a long time. According to the Bangladesh Demographic and Health Surveys (BDHS) report, the prevalence of EBF was nearly 45% in 1993–94 and 1999–2000 [24, 25], 42% in 2004 [26] and 43% in 2007 [27]. The prevalence of EBF markedly increased to 64% in the BDHS report in 2011 [28] which further declined to (55%) in the recent report of BDHS in 2014 [29]. The reasons of this rise and fall in EBF prevalence in recent times remain speculative at this point. While the BDHS collects data on national prevalence of EBF, it does not provide detailed information on factors influencing EBF, nor does it present regional rates of EBF and the causes of variation in EBF in between the regions. Furthermore, to the best of our knowledge, no elaborate study has been conducted to determine the prevalence and associated factors influencing EBF nationwide. A recent study conducted on the prevalence of EBF in a rural sub-district in Bangladesh which showed a significantly lower prevalence of EBF (36%) [22] than the national figure (55%) [29]. Therefore, it is important to sort out the local factors that influence EBF in order to implement strategies and interventions that could speed up the government efforts in improving EBF trend among mothers having infants aged 0–6 months. This study aimed to determine the prevalence and factors associated with the prediction of EBF for first 6 months of infant life in different regions of Bangladesh and the country as a whole.

## Methods

The data used in the present study was extracted from the large scale of dataset collected by Bangladesh Demographic and Health Survey (BDHS)-2014. BDHS-2014 collected socio-demographic, health, anthropometric and lifestyle information from 17,863 Bangladeshi married women aged from 15 to 49 years. The data was collected from March 24, 2014 to August 11, 2014. This is a nationally representative survey which covers all administrative regions (divisions) of Bangladesh. From the preliminary sample, the mothers were excluded for the present study who did not have children. Also excluded were mothers whose child’s age was currently less than 6 months. Besides these, some incomplete information and missing samples were also excluded from the data set and eventually there were 3541 samples for final analysis.

### Sampling

Bangladesh is divided into seven administrative divisions: Barisal, Chittagong, Dhaka, Khulna, Rajshahi, Rangpur, and Sylhet. BDHS-2014 collected data from urban and rural areas from each division using two stage stratified cluster sampling. Bangladesh Bureau of Statistics (BBS)-2011 divided Bangladesh into many small areas called enumeration areas (EA) for population and housing census. BDHS-2014 considered EA as the primary sampling unit (PSU) for their survey. In the first stage, BDHS-2014 randomly selected 600 EAs, with 207 EAs in urban and 393 in rural areas. In the second stage, they selected on average 30 households from each EA using systematic sampling. BDHS-2014 interview was successfully completed in 17,300 (99%) households. A total of 18,245 ever-married women in reproductive age were identified in these households and 17,863 were interviewed [29].

### Outcome variable

Outcome variable of this study was exclusive breastfeeding (EBF) during first six months of an infant’s life. EBF was considered only the mothers breastfeed and who did not give any supplementary food during first six months of her infant’s life. BDHS collected the duration of exclusive breastfeeding from Bangladeshi mothers by recall method [29]. The duration of EBF was divided into two groups; (i) less than 6 months (No = 0) and (ii) 6 and more months (Yes = 1) by the present authors. This categorical variable was used as a dependent variable in the present study [29].

### Independent variables

In this study we considered the following socio-economic, demographic, anthropometric and behavior variables as independent variables:

*Socio-economic variables:* Type of residence, region (division), religions, mass media access, mother’s and her husband’s education, mother’s and her husband’s occupation, and wealth index.

*Demographic variables:* Parity, early childbearing, mother’s age at first birth, last pregnancy wanted, place of delivery, mode of delivery, current age of children, current age of mother.

Anthropometric: Child’s weight at birth, initiation of breastfeeding, body mass index (BMI) of mother.

*Behavior variables:* Antenatal care, postnatal care (PNC) for mother, during first two days breastfeeding counseling.

BMI was calculated as the ratio of weight in kilograms to height in meters squared and classified according to most widely used categories of BMI for adults; these were: underweight (BMI ≤ 18.5 kg/m^2^), normal weight (18.5 < BMI < 25 kg/m^2^), overweight (25 ≤ BMI < 30 kg/m^2^) and obese (BMI ≥ 30 kg/m^2^) [30, 31].

### Statistical analysis

Chi-square (χ^2^) tests were used in this study to verify the association between EBF and some selected socio-economic, demographic, and anthropometric. Univariate and multivariate binary logistic regression models were utilized to identify influencing factors for EBF. The model fitness was tested using Hosmer and Lemeshow test, and Negelkerker R^2^. Multicollinearity problem among the predictor variables were checked by standard error (SE). If the magnitude of the SE is less than 0.5, it suggested that there is no evidence of multicollinearity problem [32]. Finally, most important predictors for EBF were determined by stepwise logistic regression model. A two-tailed *p* value of 0.05 was considered significant at the 95% CI (Confidence Interval) level. All analyses have been done by SPSS IBM version 23.

## Results

**Fig. 1:**
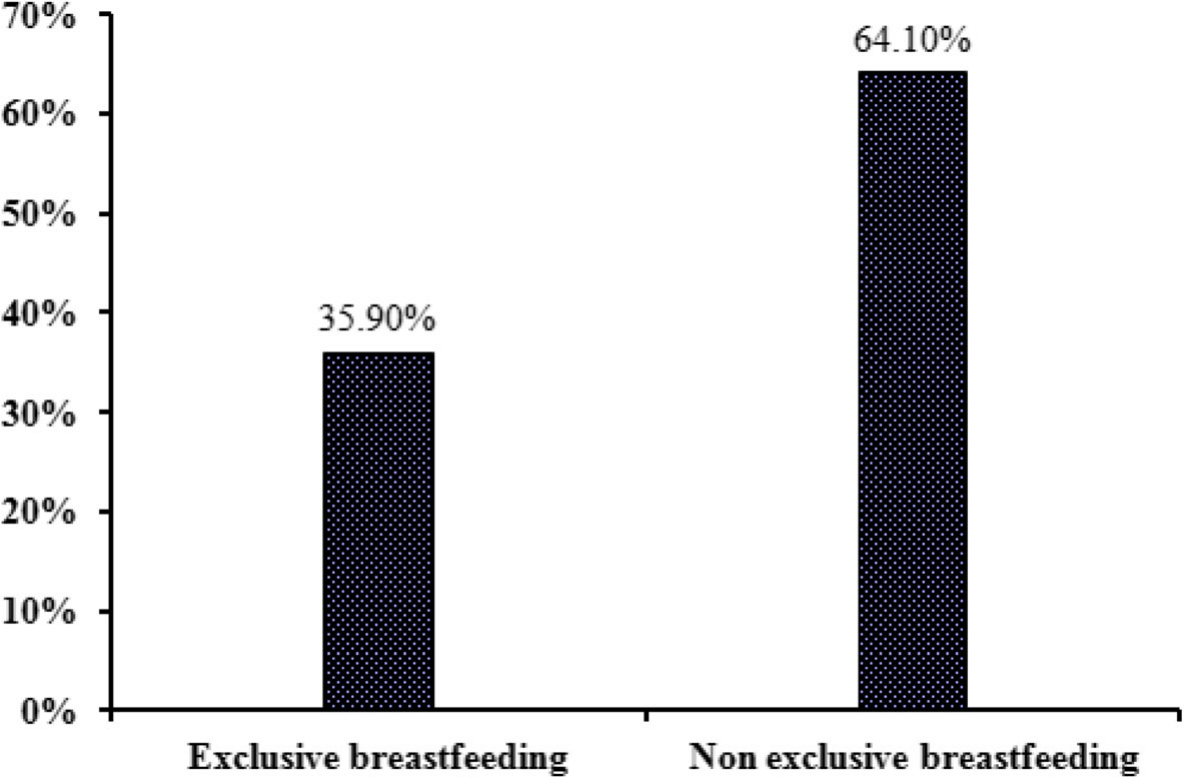
Prevalence of exclusive breastfeeding in Bangladesh during first six months of an infant’s life

A total number of 3541 ever-married and able breastfeeding mothers were analyzed for this study with mean age of 31.02 ± 9.22 years (ranging from 15 to 49). The prevalence of exclusive breastfeeding (EBF) among Bangladeshi mothers was 35.90% (Fig. 1) where the EBF in rural area was 36.3% and in urban area 35.2%.

Chi-square test demonstrated that some independent variables were significantly associated with EBF in Bangladesh. The significantly associated factors were considered as an independent variable in binary logistic models. Exclusive breastfeeding (EBF) yes = 1 was considered as a reference case and no = 0 as non-reference case for dependent variable. Table 1 represents the effect of socio-economic and demographic factors on EBF among Bangladeshi mothers. The coefficient and adjusted odds ratio (AOR) of multivariable binary logistic regression model demonstrated that the mothers who lived in Sylhet division were more likely to breastfeed her children than those who lived in Dhaka [AOR = 0.40; 95% CI: 0.20–0.80, *p* < 0.01] and Rajshahi divisions [AOR = 0.36; 95% CI: 0.18–0.74, *p* < 0.01]. Primary educated mothers were more likely to practice EBF [AOR = 2.28; 95% CI: 1.05–4.93, *p* < 0.05] than higher educated mothers. Mothers who were housewives [AOR = 1.20; 95% CI: 1.02–1.42, *p* < 0.05] were more likely to practice EBF than worker/business/service holder mothers and those whose husbands were service holders had 1.64 times [95% CI: 1.02–2.62, *p* < 0.05] higher chances of EBF than business/other professions. Current age of children were found as a significant (AOR = 0.01, 95% CI: 0.00–0.01; *p* < 0.001) predictor for EBF (Table 1).

**Table 1:** Effect of socio-economic and demographic factors on exclusive breastfeeding during first 6 months of an infant’s life in Bangladesh

Characteristics	Unadjusted Odds Ratio (OR)	95% CI of OR	*p*-value	Adjusted OR	95% CI of OR	*p*-value
Region			0.008			
Barisal	0.94	0.72 to 1.22	0.641	0.96	0.44 to 2.13	0.928
Chittagong	0.94	0.74 to 1.18	0.586	0.65	0.33 to 1.27	0.211
Dhaka	0.67	0.52 to 0.85	0.001	0.40	0.20 to 0.80	0.009
Khulna	0.91	0.70 to 1.19	0.506	0.71	0.33 to 1.52	0.374
Rajshahi	0.75	0.57 to 0.97	0.031	0.36	0.18 to 0.74	0.006
Rangpur	0.99	0.77 to 1.29	0.983	0.71	0.33 to 1.50	0.366
Sylhet^r^	–	–	–	–	–	–
Mother age			< 0.001			
15–19	0.34	0.25 to 0.46	< 0.001	1.10	0.38 to 3.13	0.865
20–24	0.51	0.38 to 0.68	< 0.001	0.80	0.32 to 1.99	0.638
25–29	0.65	0.49 to 0.88	0.004	1.21	0.51 to 2.84	0.669
30–34	0.68	0.50 to 0.94	0.019	0.79	0.34 to 1.84	0.589
35 and more^r^	–	–	–	–	–	–
Mother education			0.033			
Illiterate	1.47	1.11 to 1.95	0.007	1.87	0.73 to 4.76	0.190
Primary	1.23	0.96 to 1.58	0.104	2.28	1.05 to 4.93	0.037
Secondary	1.14	0.90 to 1.44	0.276	1.75	0.95 to 3.24	0.074
Higher^r^	–	–	–	–	–	–
Father education			0.003			
Illiterate	1.38	1.10 to 1.73	0.005	1.16	0.54 to 2.51	0.703
Primary	1.06	0.85 to 1.32	0.612	0.98	0.50 to 1.92	0.955
Secondary	1.00	0.81 to 1.25	0.983	1.24	0.69 to 2.25	0.471
Higher^r^	–	–	–	–	–	–
Mother occupation						
Housewife	0.76	0.65 to 0.89	0.001	1.20	1.02 to 1.42	0.049
Worker/business/service holder^r^	–	–	–	–	–	–
Father occupation			0.041			
Farmer/worker	1.25	1.05 to 1.49	0.012	1.36	0.84 to 2.22	0.214
Service holder	1.18	0.98 to 1.42	0.079	1.64	1.02 to 2.62	0.039
Business/others^r^	–	–	–	–	–	–
Mass media access						
Yes	1.17	1.02 to 1.35	0.030	1.54	0.94 to 2.50	0.084
No^r^	–	–	–	–	–	–
BMI for mother			0.004			
Underweight	0.65	0.42 to 1.01	0.056	1.20	0.48 to 3.03	0.696
Normal weight	0.87	0.57 to 1.33	0.533	1.63	0.69 to 3.85	0.267
Overweight	0.86	0.55 to 1.35	0.510	1.07	0.44 to 2.61	0.887
Obese^r^	–	–	–	–	–	–
Total children ever born			< 0.001			
One children	0.67	0.54 to 0.83	< 0.001	0.73	0.31 to 1.71	0.469
2–3 children	0.82	0.67 to 1.01	0.060	1.26	0.64 to 2.50	0.508
4 and more children^r^	–	–	–	–	–	–
Delivery mode for last pregnancy						
Non-caesarean	1.25	1.06 to 1.48	0.007	1.08	0.58 to 2.00	0.809
Caesarean^r^	–	–	–	–	–	–
Delivery place						
Home	1.28	1.11 to 1.48	0.001	1.22	0.68 to 2.16	0.506
Hospital/clinic^r^	–	–	–	–	–	–
Antenatal care						
Yes	1.33	1.13 to 1.56	0.001	0.86	0.51 to 1.45	0.580
No^r^	–	–	–	–	–	–
Postnatal care for mother						
Yes	1.31	1.14 to 1.51	< 0.001	1.17	0.62 to 2.21	0.619
No^r^	–	–	–	–	–	–
Breast feeding counseling during first two days						
Yes	1.17	1.02 to 1.34	0.026	1.22	0.73 to 2.05	0.443
No^r^	–	–	–	–	–	–
Current age of children						
6 month to 1 year	0.01	0.00 to 0.01	< 0.001	0.01	0.00 to 0.01	0.000
More than one year^r^	–	–	–	–	–	–
Hosmer and Lemeshow test		χ^2^-value = 7.798		*p*-value = 0.454		
Nagelkerke R^2^ value =0.885						

r = reference case

Hosmer and Lemeshow test (Chi-square value = 7.798) showed that the observed and estimated values were very close (*p* > 0.05), suggesting the selected binary multiple logistic model was well-fitted for the data. Moreover, Nagelkerke R^2^ demonstrated that our model was able to explain the variation of dependent variable by 88.5% (Table 1).

## Discussion

Exclusive breastfeeding (EBF) practice during the first six months of infant’s life is the most effective intervention for providing balanced nutrition and for the prevention of child mortality and morbidity. In this study, we observed that the overall prevalence of EBF practice among Bangladeshi mothers was 35.9%. The rate of EBF practice was lower in this study than the BDHS report 2016 which showed an overall EBF rate of 55% in Bangladesh. The prevalence of EBF in Bangladesh according to this study was higher than that reported in some other countries such as Egypt (9.7%) [33], India (Tamil Nadu, 34%) [34], Saudi Arabia (Al-Hassa, 24.4%) [35] and the USA (16.8%) [36]. However, the prevalence of EBF was found higher in some other parts of the world such as Malaysia (Peninsular, 43.1%) [19], Arbaminch Southern Ethiopia (46.5%) [37], Bahir Dar city of Northwest Ethiopia (50.3%) [23], Debre Markos of Northwest Ethiopia (60.8%) [20], Western India (61.5%) [38] and the Goba district of South East Ethiopia (71.3%) [39]. The variations persisting in EBF rate in different regions worldwide might be due to cultural, economic and socio-demographic differences across areas. Besides, all the countries probably are not focusing on enhancing the EBF rate with the same intensity which may also contribute to the discrepancy. The other possible reasons for the variation in EBF practice found in different studies may be the different methods used for measuring EBF. In this study, recall method was used for assessing EBF. In a study in Bahir Dar city of Northwest Ethiopia, Seid et al. used ‘since birth dietary recall’ method [23] which is not a standard method of determining EBF. A ‘seven day self-recall’ method was used to assess EBF in some other studies including Peninsular Malaysian study [19] and Debre Markos (Northwest Ethiopia) study [20]. Moreover, a Ghanaian study demonstrated a significant differences in EBF determined by ‘24-h recall’ method and ‘since birth dietary recall’ method (70.2% versus 51.6%) [40], which further supports of the discrepancy in EBF rate on the basis of determining EBF methods.

In Bangladesh, there are seven major administrative regions (divisions). Besides the prevalence of overall EBF in Bangladesh, this study also analyzed the regional rate of EBF practice among the mothers of the representative regions. Among the regions, the prevalence of EBF was found highest in Sylhet and Rangpur divisions followed by Barisal, Chittagong, Khulna, Rajshahi, and Dhaka divisions. The literacy rate of women in both Sylhet and Rangpur divisions is lower than that of other administrative regions of Bangladesh which could be an attributing factor for higher EBF rate in these regions [41, 42].

Our study identified several other socio-demographic factors associated with EBF in Bangladesh. Mothers’ age has been found as a major determining factor significantly associated with EBF. Younger mothers were less likely to adhere to the EBF practice and the EBF rates increased among the mothers with the increase in age. Mothers aged between 15 to 19 years were almost 0.34 times less likely to exclusively breastfed their infants than their counter part mothers aged 35 years and above (*p* < 0.001). This finding is consistent with the study conducted in Debre Berhan District and Hareri Regional State of Ethiopia [21, 43]. This could be due to the fact that younger mothers may have lack of awareness and knowledge of breastfeeding. Moreover, they do have more job opportunities than older mothers and lack the time to exclusively breastfed their infants.

Mothers’ education and occupation were found inversely proportional to EBF practice in many studies. In this study, illiterate mothers were more likely to provide EBF to their infants and the practice rate of EBF was significantly reduced with the increase in mothers’ educational status. These findings are in agreement with the findings of Al-Hassa, Saudi Arabia [35], Arbaminch Ethiopia [37], Bahir Dar district Ethiopia [23], Debre Berhan district Ethiopia [21], Debre Markos district Ethiopia [20], Goba district Ethiopia [39], Peninsular Malaysia [19] and Tamil Nadu India [34]. This could be explained as the fact that educated mothers have better job opportunities in Bangladesh and they are likely to join services. Therefore, educated and employed mothers may not have or may not be able to manage sufficient time during working hours to breastfeed their infants. However, these results do not essentially mean that education and employment cause failure to EBF. Additional factors such as weaning as a part of preparation to get back to work, maternal fatigue and the pressure of fulfilling the demands of work may also contribute to this issue. In Bangladesh, only working mothers in government organizations, but not in non-government organizations, are given six months of maternity leave. Moreover, the amenities of breastfeeding in most of the work places are quite unacceptable to breastfeed. These may lead the educated-employed mothers not to breastfeed their infants as compared to illiterate and housewife mothers.

In line with mothers’ education, fathers’ education has also been found to be significantly associated with EBF practice according to simple univariate analysis. Mothers who have illiterate husbands (illiterate fathers of the infants) were more likely to breastfeed than mothers who have educated husbands. Fathers’ employment status also was found to influence the EBF practice in this study. This may be due to the fact that educated fathers are mostly involved in service and business, and cannot manage enough time to support their wives. This can also be attributed to the fact that many of the educated fathers live away from their family to continue their service in the organizations they are employed in. It was reported that EBF was more common among mothers with supportive husbands than the mothers having non-supportive husbands [19].

In this study, we found antenatal care (ANC) was significantly associated with EBF practice among Bangladeshi mothers from univariate analysis while multivariate analysis showed this factor as insignificant. Mothers who received ANC were 1.33 times more likely to provide EBF to their infants compared to those mothers who did not receive the ANC. This could be due to the ANC programs that include breastfeeding counseling which in turn improves breastfeeding knowledge of mothers and motivates them to exclusively breastfed their infants. Breastfeeding counseling during ANC was also identified as a significant factor associated with EBF practice in some other studies from Egypt [33], Nigeria [44], Debre Markos, Ethiopia [20] and Arbaminch, Ethiopia [37].

*Postnatal care* (PNC) is the health service given to the mother and the newborn child immediately after birth and for the first six weeks of baby’s life. PNC, that includes infant feeding counseling, was also recognized as a significant factor associated with EBF. In simple analysis, the rate of EBF was 1.31times higher among mothers who received PNC than the mothers who did not receive PNC. Those mothers who received breastfeeding counseling for first two days during PNC had a much higher (1.17 times) chance of practicing EBF to their babies than those who did not receive counseling according to univariate analysis. The observed association between breastfeeding counseling and EBF prevalence is in line with the study findings from Bahir Dar, Ethiopia [23], Debre Berhan, Ethiopia [21], Debre Markos, Ethiopia [20] and Western India [38]. PNC for baby within two months also significantly increased the rate of EBF practice of their mothers compared to the mothers whose babies did not receive PNC care within two months period. This could be the result of the increased health facilities and services of the trained health professionals especially midwives who teach mothers the proper breast feeding practices for their infant and young child.

Mass media access was also indicated as a significant factor associated with EBF practice in this study. From simple analysis the study found that mothers who had frequent access to mass media had a higher adherence to practice EBF for the first six months of infants’ life than those mothers who did not have access. In a recent study, mass media (radio or television) was reported as the second best source of information for mothers on EBF (30.4%) only after the health professionals (90.5%) [21].

Higher rates of EBF are common among mothers having multiple children. This study also found the lowest EBF rates among mothers with only one child ever born and the rates of EBF practice were increased among mothers with increasing number of children. A lack of knowledge and experience of appropriate breastfeeding for mothers with their first child may cause the discontinuation of EBF. This finding is further supported by the studies pursued in Peninsular Malaysia and Hong Kong [19, 45]. The authors reported that mothers with their first child were in low self confidence, less knowledgeable and unskillful in breastfeeding their infants.

A significant correlation was found between EBF practice and the place of delivery. In this study, mothers who gave birth to their children at home were more adherent to practice EBF compared to mothers who delivered their children in health institutions e.g. hospitals and clinics. However, in contrast to this finding, studies from Ethiopia and Tanzania showed a higher prevalence of EBF practice among mothers who delivered in health institutions than their counter parts who delivered at home [21, 23]. Higher rates of EBF practice among Bangladeshi mothers who gave birth in home was rather unusual. This unexpected finding could be explained as the fact that most of the mothers in Bangladesh are till now used to giving birth to their babies in home with the help of midwives. They get admitted into health care institutions only when they face any complication and the expected mothers are in a critical situation. Even in many cases, many pregnant women were reported to get admitted into hospitals once they failed to give birth to the baby at home. Majority of these women may need to undergo surgery for the delivery of the baby which may contribute to reduced EBF practice rate among mothers delivering their babies in health care institutions in Bangladesh. This speculation is further supported in this study by the fact that mothers who had non-caesarean delivery were more likely to exclusively breastfed their infants compared to mothers who had caesarean delivery (37.1% vs 32.0%). A similar finding was also reported in studies from a sub-district of Bangladesh and Ethiopia [22, 23]. This could be due to the fact that mothers may face health complications during/after caesarean section, they may need some longer time to recover from caesarean section related pain and discomfort which in turn may present mothers from practicing EBF. It was also suggested that cesarean delivery may result in delayed milk production [46, 47] which may also contribute to lower rate of EBF practice among the cesarean mothers.

Among the socio-demographic characteristics considered in this study, the place of residence, religion, wealth quintile, early childbearing, wanted last pregnancy, initial breastfeeding, age of child, sex of child, birth size of child and parity did not show a statistically significant correlation with EBF practice. In other studies, younger age child [23] and female child [44] were found to have a positive association with mothers’ EBF practice. These variations could be due to the differences in existing socio-cultural rituals on child feeding and sex preferences among the study populations.

### Limitations

This study has several potential limitations. Since this is a cross-sectional study, it is difficult to establish a causal relationship between the determinant factors and EBF. Last night self-recall method was used for assessing EBF where as longitudinal study is more effective. Despite the above shortcomings, the findings of this study will contribute to understanding and to identifying the factors associated with EBF practice in Bangladesh.

## Conclusions

While breastfeeding especially EBF is recommended for proper growth and development of the newborn infants, the prevalence of EBF up to first six months of the infant’s life in Bangladesh is reasonably low. This study revealed a number of socio-demographic factors such as mothers’ age, mothers’ education and occupation, fathers’ education and occupation, mass media access, total number of children ever born, place of delivery, mode of delivery, ANC, PNC for mothers and breastfeeding counseling were independently and significantly associated with EBF practice in Bangladesh. Interventions that need to be considered to improve EBF practice include increasing media coverage regarding the awareness programs of breastfeeding, establishing breastfeeding-friendly working environment for working mothers and work-site day care centers for infants, establishing maternal health clinics and health extension programs throughout the country so that more number of pregnant women and mothers can receive appropriate health services, strengthening infant feeding counseling both at the community and institutional levels, discouraging home delivery, extending maternity leave up to the first six months after delivery and introducing paternity leave at least for first one or two months of infants’ delivery. Initiatives should be taken for the proper execution of the recommended interventions which would be able to significantly increase the EBF practice among mothers in Bangladesh.

## Abbreviations

ANC: Antenatal Care
AOR: Adjusted Odds Ratio
BBS: Bangladesh Bureau of Statistics
BDHS: Bangladesh Demographic and Health Survey
BMI: Body Mass Index
CI: Confidence Interval
EA: Enumeration Area
EBF: Exclusive breastfeeding
HIV: Human Immune Deficiency Virus
IBM: International Business Machines
IQ: Intelligence quotient
NIPORT: National Institute of Population Research and Training
PNC: Postnatal Care
PSU: Primary Sampling Unit
SE: Standard Error
SPSS: Statistical Package for the Social Science Software
USA: United State of America
Vs: Versus
WHO: World Health Organization
χ^2^: Chi-Square
